# Overview of Cyanide Poisoning in Cattle from *Sorghum halepense* and *S. bicolor* Cultivars in Northwest Italy

**DOI:** 10.3390/ani14050743

**Published:** 2024-02-27

**Authors:** Stefano Giantin, Alberico Franzin, Fulvio Brusa, Vittoria Montemurro, Elena Bozzetta, Elisabetta Caprai, Giorgio Fedrizzi, Flavia Girolami, Carlo Nebbia

**Affiliations:** 1 Istituto Zooprofilattico Sperimentale del Piemonte, Liguria e Valle d’Aosta (IZSPLV), Via Bologna 148, 10154 Turin, Italy; stefano.giantin@izsto.it (S.G.); alberico.franzin@izsto.it (A.F.); fulvio.brusa@izsto.it (F.B.); vittoria.montemurro@izsto.it (V.M.); elena.bozzetta@izsto.it (E.B.); 2 National Reference Laboratory for Natural Toxins in Food and Feed, Istituto Zooprofilattico Sperimentale della Lombardia e dell’Emilia Romagna (IZSLER), Via Pietro Fiorini 5, 40127 Bologna, Italy; elisabetta.caprai@izsler.it (E.C.); giorgio.fedrizzi@izsler.it (G.F.); 3 Department of Veterinary Sciences, University of Turin, Largo Paolo Braccini 2, 10095 Grugliasco, Italy; flavia.girolami@unito.it

**Keywords:** *Sorghum bicolor*, *Sorghum halepense*, dhurrin, cyanide poisoning, cattle, drought

## Abstract

**Simple Summary:**

Both wild (*Sorghum halepense*) and cultivated Sorghum (*Sorghum bicolor*) species are commonly used for animal feeding. However, sorghum plants naturally produce dhurrin, a cyanogenic glycoside releasing cyanide; this extremely toxic molecule potentially causes lethal toxicoses, particularly in ruminants. This was the case in a number of farms located in Northwest Italy during August 2022, under weather conditions (drought, tropical temperatures) known to increase plants’ dhurrin content. Sixty-six bovines died after grazing Sorghum pastures (*Sorghum bicolor* or *Sorghum halepense*) or being fed with Sorghum-containing hay (*Sorghum halepense*). The reported clinico-pathological findings clearly indicated cyanide poisoning, and chemical analysis revealed high concentrations of dhurrin in the plant materials. The successful management of such toxicosis should rely on the prompt removal of the contaminated fodder and the administration of the antidote sodium thiosulphate. Dhurrin content should be carefully monitored, particularly in the hot season, and both farmers and veterinarians should be made aware of the risks associated with feeding cattle even with cultivated Sorghum, particularly if grown under adverse climatic conditions.

**Abstract:**

*Sorghum* plants naturally produce dhurrin, a cyanogenic glycoside that may be hydrolysed to cyanide, resulting in often-lethal toxicoses. Ruminants are particularly sensitive to cyanogenic glycosides due to the active role of rumen microbiota in dhurrin hydrolysis. This work provides an overview of a poisoning outbreak that occurred in 5 farms in Northwest Italy in August 2022; a total of 66 cows died, and many others developed acute toxicosis after being fed on either cultivated (*Sorghum bicolor*) or wild Sorghum (*Sorghum halepense*). Clinical signs were recorded, and all cows received antidotal/supportive therapy. Dead animals were subjected to necropsy, and dhurrin content was determined in Sorghum specimens using an LC–MS/MS method. Rapid onset, severe respiratory distress, recumbency and convulsions were the main clinical features; bright red blood, a bitter almond smell and lung emphysema were consistently observed on necropsy. The combined i.v. and oral administration of sodium thiosulphate resulted in a rapid improvement of clinical signs. Dhurrin concentrations corresponding to cyanide levels higher than the tolerated threshold of 200 mg/kg were detected in sorghum specimens from 4 out of 5 involved farms; thereafter, such levels declined, reaching tolerable concentrations in September–October. Feeding cattle with wild or cultivated Sorghum as green fodder is a common practice in Northern Italy, especially in summer. However, care should be taken in case of adverse climatic conditions, such as severe drought and tropical temperatures (characterising summer 2022), which are reported to increase dhurrin synthesis and storage.

## 1. Introduction

*Sorghum* is a genus of plants belonging to the family of *Poaceae*, widely used as forage crop as well as human food and for biofuel production. Globally, the most cultivated species is *Sorghum bicolor* (L.) Moench, also known as broomcorn or great millet; this species is particularly widespread in the Americas and Africa, which, in 2017–2021, accounted together for more than 80% of total production. In 2021, Europe produced 1.9% of global Sorghum, with France being the highest-producing country at 386,040 t [1]. In Italy, the second highest-ranking European producer, the Sorghum yield reached 242,855 t in 2023, making it the fourth cereal after wheat, corn and rice [2]. Some wild species are also exploited for animal feeding, such as the widespread *Sorghum halepense* (L.) Pers., commonly referred to as Johnson grass, originating from the Mediterranean and Western Asia regions and now reported as an invasive weed across all continents [3].

When used as feed, Sorghum must be managed with particular caution because of a cyanogenic glycoside called dhurrin ((S)-4-Hydroxymandelonitrile β-d-glycoside) [4], which is synthesised as a secondary metabolite in its tissues. This molecule contains a cyanide group (CN^−^) that can be released upon hydrolysis and is extremely toxic to all eukaryotic cells. CN^−^ inhibits cellular respiration by binding to the Fe^+++^ of cytochrome oxidase, rendering cells unable to utilise molecular oxygen and ultimately to synthetise ATP [5]. The rumen microbiota is able to rapidly hydrolyse dhurrin, further accelerating cyanide release and therefore making ruminants much more sensitive to CN^−^ than monogastric species [6,7]. Such rapid and massive CN^−^ release can cause severe, often lethal, poisonings in ruminants, particularly upon the ingestion of large amounts of fodder with high dhurrin content [6].

In Sorghum plants, dhurrin is produced especially during early growth phases [5,8]. Thanks to this glycoside, *Sorghum* species are quite resistant to herbivores, including insects [9]. Mature plants generally contain a lower amount of dhurrin and are therefore considered safe for animal feeding; however, dhurrin content is reported to increase under the following conditions [10]:

- Prolonged drought, frost, wilting, chewing and any other condition causing plant cell injury;
- Massive herbicide treatments;
- Extensive use of nitrogen-based fertilisers.

When used as feed, Sorghum can be directly grazed by animals or harvested for green forage, silage and hay production. Generally, the ensiling process leads to a dispersal of CN^−^ from plant tissues. Still, in some cases, high CN^−^ concentrations can remain in plants that have undergone rapid desiccation and subsequent conservation in large bales [9]. Because of the CN^−^ poisoning potential, Sorghum harvesting and use require cautious management in order to minimise poisoning risks, with special attention when used for feeding ruminants. Young leaves and new shoots, including the sprouts, are the most dangerous parts, as they can concentrate large amounts of dhurrin [6].

Although Sorghum toxicity has long been known, no poisoning cases in bovines have been reported in Europe in recent decades [11,12], with the exception of two cases in Spain quoted in a review on plant poisoning [13]. A search of the grey literature also revealed no results in Europe, but there were several cases in both the Americas and Australia [14]. Likewise, data on Sorghum poisonings were found—both in scientific databases and through online search engines—in extra-European countries, especially in semi-arid regions of South America [15,16,17] and India [18,19,20], where Sorghum cultivation for fodder purposes is common.

In August 2022, 66 bovines died in Piedmont (a region in Northwest Italy) after being exposed to the *S. bicolor* × *S. sudanense*—i.e., *S. bicolor* ssp*. sudanense* (P.) Stapf—cultivar called Suzy [21] or to forage containing *S. halepense*. The aim of this study is to provide a detailed overview of this outbreak, with special emphasis on the diagnosis and the therapeutic management of this toxicosis. Results of dhurrin concentration monitoring from August to November 2022 in both cultivated and wild *Sorghum* samples from the affected farms and elsewhere are also presented. A short preliminary report of the outbreak has been published in Italian in 2023 [22].

### 1.1. Poisoning Cases (August 2022)

**Figure 1:**
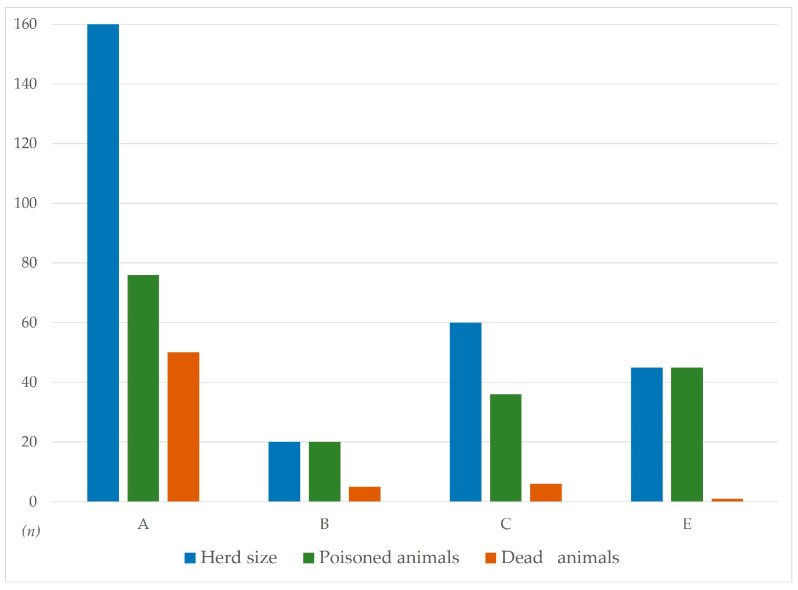
Epidemiological data (herd size, morbidity and mortality rate) of the five reported poisoning cases: A—Sommariva del Bosco; B—Moretta; C—Bra; E—Cossato. Case D (Asti) is not shown due to the lack of reliable information.

Five outbreaks of Sorghum poisoning occurred in August 2022 in Piedmont. Figure 1 shows the epidemiological data concerning the poisoning cases.

#### 1.1.1. Case A—6th of August: Sommariva del Bosco (Cuneo)

**Figure 2:**
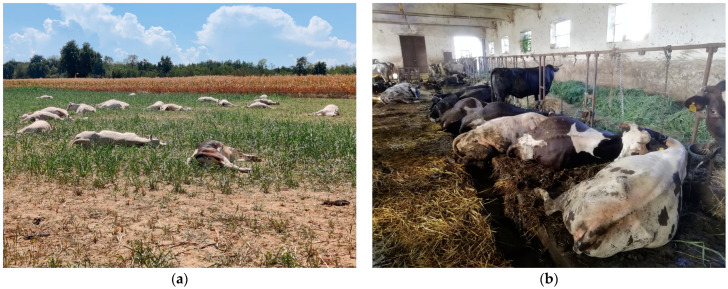
Case A—Sommariva del Bosco (**a**) and B—Moretta (**b**): poisoned/dead animals in lateral recumbency, mostly on the right side.

A herd of 160 cows, mainly of the Piedmontese breed, was allowed free access to a field entirely cultivated with the *S. bicolor* × *S. sudanense* cultivar Suzy. As the animals were hungry due to overnight fasting, they rapidly ingested Sorghum plants, specifically sprouts with a height range of 30–45 cm. Around half of the animals were poisoned; forty-six of them rapidly died 20–30 min after the ingestion (Figure 2a), while in four further individuals, death ensued in the following hours. Most of the dead individuals were pregnant. The surviving ones were immediately moved away. Based on the clinical picture, the sudden deaths and the gross lesions (see below), cyanogenic glycoside poisoning was promptly suspected.

#### 1.1.2. Case B—11th of August: Moretta (Cuneo)

A group of 20 adult mixed-breed cows and bulls (mainly Friesian × Piedmontese or other meat breeds) housed in tie stalls were fed green chop (fresh forage) mainly composed of *S. halepense*. This is a common farming practice in Piedmont, especially during the warm season, when green and high-quality forage is scarce. All the affected animals were lactating cows aged more than 3 years; five of them suddenly died after being offered the contaminated feed (Figure 2b). The forage was promptly removed from the troughs after the onset of the clinical signs of poisoning.

#### 1.1.3. Case C—11th of August: Bra (Cuneo)

Sixty adult cows of the Piedmontese breed housed in a free-stall barn were fed green chop, mainly consisting of Johnson grass. Poisoning signs were noticed during the subsequent night in thirty-six individuals; four of these suddenly died after grass ingestion, while in two further individuals, death ensued few days later. As in case B, the forage was removed after the first symptoms, and no further mortality was recorded.

#### 1.1.4. Case D—12th of August: Asti

This case occurred in a cow–calf operation farm consisting of about 60 heads of the Piedmontese breed (cows and calves) housed in a free-stall barn. By day, the animals were allowed to graze on pastures in proximity to the farm for most of the year. Four cows died after ingestion of *S. halepense*, which was found to contaminate the pasture. This episode was tardily and poorly reported to the veterinarians, such that it was not possible to collect reliable epidemiologic information.

#### 1.1.5. Case E—25th of August: Cossato (Biella)

The farm’s characteristics were similar to those from case D, i.e., a cow–calf operation farm with about 45 head, mainly of the Piedmontese breed (but also meat crossbreds). For most of the year, animals were free to graze on pastures surrounding the farm. All cows showed the typical signs of cyanide poisoning, mainly respiratory distress and a tendency toward recumbency; overall, symptoms were less severe than in cases A, B and C, resulting in the loss of only one cow. Also in this case, the cause of poisoning was pasture contamination with *S. halepense*.

## 2. Materials and Methods

### 2.1. Necropsies and Histological Analysis

Due to unfavourable conditions (high external temperatures and the limited availability of veterinarians), necropsies were performed on only a few animals (*n* = 6 in total) directly at the farms. Heart, lung, brain, liver, kidney, spleen, reticulum, rumen, omasum, abomasum and intestine samples were collected, fixed in 10% buffered formalin (4% formaldehyde), dehydrated and embedded in paraffin wax blocks. Each sample was then sectioned at 4–5 µm-thickness, mounted on glass slides and stained with haematoxylin and eosin to reveal histopathological alterations. Slides were examined by two independent veterinary pathologists.

### 2.2. Sorghum Sample Collection

**Figure 3:**
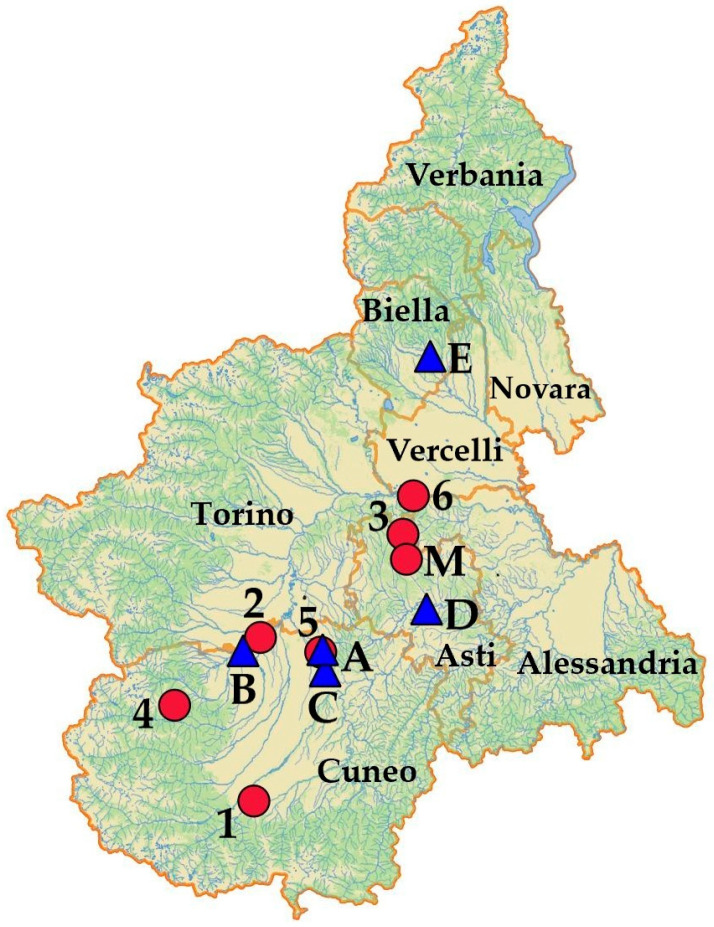
Map of the Piedmont region showing the locations of the poisoning cases (blue triangles: A—Sommariva del Bosco, B—Moretta, C—Bra, D—Asti, E—Cossato) and the other farms selected for sampling of either cultivated Sorghum (red circle: M—Montechiaro d’Asti) or wild Sorghum (red circles: 1—Cuneo, 2—Faule, 3—Montiglio Monferrato, 4—Sampeyre, 5—Sanfrè, 6—Verrua Savoia). All the samples (*n* = 57) were collected from August to November 2022. Orange lines indicate province borders.

To confirm the suspicion of cyanogenic glycoside poisoning, samples of Sorghum to which the cattle were exposed were collected at each farm involved in the outbreak (Figure 3) and submitted for dhurrin determination (see below). It was also decided to collect and analyse additional specimens of both wild and cultivated Sorghum in order to measure dhurrin content in plants from different areas of the Piedmont region. In particular, the selection process was based on three main factors:

- Areas where strong drought conditions occurred [23];
- Requests for dhurrin analysis from a number of worried farmers;
- Financial resources.

In addition, in certain instances, samples were collected from different plant portions and at diverse growth stages. 

As regards cultivated Sorghum, the *S. bicolor* × *S. sudanense* cultivar Suzy was involved in case A. Two cultivars, *S. bicolor* ssp*. drummondii* Piper and *S. bicolor* × *S. sudanense* Sudal [24], were then sampled from a farm in Montechiaro d’Asti (Asti), which was experiencing similar drought conditions to the farm in case A; Sorghum had not yet been harvested due to the severe outbreak that had occurred in Sommariva del Bosco.

Common Johnson grass, which is frequently used as fodder by Piedmontese farmers, was the cause of poisoning cases B, C, D and E. Further sites for *S. halepense* sampling were Verrua Savoia (Torino province), Montiglio Monferrato (Asti province), Cuneo, Faule, Sampeyre and Sanfrè (Cuneo province).

One pooled sample composed of a minimum of 500 g of fresh plant materials was collected randomly from different areas inside pasture fields or directly taken from the green forage offered to animals in the stalls. Additionally, in one case (A), the rumen content was collected from a dead cow. All sampling activities were completed from August to November 2022.

### 2.3. Dhurrin Determination

Samples were analysed using an in-house liquid chromatography–tandem mass spectrometry (LC–MS/MS) method at the National Reference Laboratory for Plant Toxins, Food Chemical Department of Istituto Zooprofilattico Sperimentale della Lombardia e dell’Emilia Romagna (IZSLER), located in Bologna. Samples were ground into flour, and 1 ± 0.1 g of each one was extracted with 6 mL of aqueous methanol (80%). The sample was shaken vigorously for 30 s and placed in an ultrasonic water bath for 15 min. The mixture was centrifugated for 5 min at 4000× *g*, and the supernatant was collected in another tube. This extraction was repeated twice, and the supernatant was combined and made up to a volume of 20 mL with water. Thereafter, 1 mL of solution was evaporated to dryness under a stream of nitrogen at 40 °C. The residue was dissolved in 0.5 mL of 10% methanol in aqueous solution, diluted and analysed by using LC–MS/MS. 

The LC–MS/MS analysis was performed on a XEVO Tq-XS Acquity ultra-performance liquid chromatograph (UPLC) I Class Plus from Waters (Milford, MA, USA). Chromatographic separation was achieved on an Acquity UPLC C8 BEH column measuring 100 mm × 2.1 mm, 1.7 µm (Water Corporation, Milford, MA, USA). Data acquisition and processing were carried out using MassLynx software v. 4.2. SCN1012. Mobile phase A consisted of 0.1% formic acid in water/acetonitrile (95:5, *v*/*v*), and mobile phase B consisted of 0.1% formic acid in acetonitrile. The following gradient was used: 0–0.5 min, isocratic 2% B; 0.5–4 min, linear gradient 2–50% B; return to initial conditions in 0.5 min and hold for 1 min. The total run time was 6 min. The flow rate was 0.4 mL/min. The injection volume was set at 5 µL. The ESI source operated in positive ionisation mode with the following instrumental parameters: capillary voltage of 0.5 kV, cone voltage of 40 V, source temperature of 120 °C and desolvation temperature of 600 °C. The conditions of ionisation and fragmentation were identified by continuous infusion of tuning solutions and gradual adjustment of the parameters. According to SANTE/12089/2016 [25], dhurrin was identified by the retention time, ion fragments and ion ratio. LC–MS/MS parameters for dhurrin determination (retention time, precursor ions, daughter ions and fragmentation conditions) are shown in Table S1. The retention time was within ±0.2 min of the reference peaks. The peaks showed similar shapes and overlapped with each other. The ion ratio was within ±30% of the average of the calibration standards from the same sequence. The peaks were within the linear range of the detector with an S/N ≥ 3 [26]. The LC–MS/MS method’s selectivity was evaluated by acquiring the data in MRM mode and monitoring one precursor ion and two daughter ions for each molecule [25].

A multi-level calibration curve with concentration levels from lowest to highest (0.2–0.5–1–2.5–5–10–15 µg/mL) was prepared in 10% methanol in aqueous solution. A correlation coefficient (R^2^) ≥ 0.99 and a normal distribution of residuals lower than 20% were achieved in every analytical batch. The calibration curve, a representative chromatogram of dhurrin reference material (2.5 µg/mL) and a chromatogram of a blank and a contaminated Sorghum sample are shown in Figures S1–S4.

The limit of quantification (LOQ) of dhurrin in feed was 50 mg/kg, corresponding to 4.3 mg/kg hydrogen cyanide (HCN), i.e., cyanide. It has been evaluated under conditions of accuracy and precision, verifying the signal-to-noise ratio to be at least equal to 10. The recovery % (70–120) of the quality control spiked at LOQ was in line with the guidance document on performance criteria of the European Union Reference Laboratory for Mycotoxin and Plant Toxins [26]. According to EFSA [27], 1 g of dhurrin has an HCN potential of 86.7 mg, representing the total amount of HCN released under conditions of complete hydrolysis of the present dhurrin. For the sake of simplicity, in this paper, the HCN potential is referred to as HCN/cyanide concentration.

### 2.4. Clinical Picture

Poisoned bovines showed multiple symptoms, with variable distribution among individuals. Many cows were found in sternal or lateral recumbency, mainly on the right side. Respiratory distress was observed in most of the poisoned animals, consisting of tachypnoea, dyspnoea, panting and gasping. Several cows also displayed stupor, convulsions and muscle twitching with vocalisations (mooing). Sialorrhoea was an additional common symptom among poisoned bovines. Moreover, light to moderate tympanism was detected in a few individuals. Hyperthermia, nystagmus, mydriasis and wheezes were occasionally observed.

### 2.5. Therapeutical Protocols

Table 1 depicts the treatment performed in each case and the relative success rate.

#### 2.5.1. Case A

Although, as mentioned above, a cyanogenic glycoside poisoning was suspected, it was difficult to find the proper remedies also because this outbreak happened during the weekend. Thirty animals were treated intravenously with a mix of rehydrating solutions (Ringer’s lactate, physiological and glucose solutions), coupled with 60 mL of the multivitamin Dobetin B1^®^ (cyanocobalamin 1 mg/mL, thiamine hydrochloride 100 mg/mL). Considered the hot external temperature (over 38 °C), the cows were also cooled down by spraying with water taken from the mobile drinking troughs. Twenty-six of the treated animals survived.

#### 2.5.2. Case B

**Figure 4:**
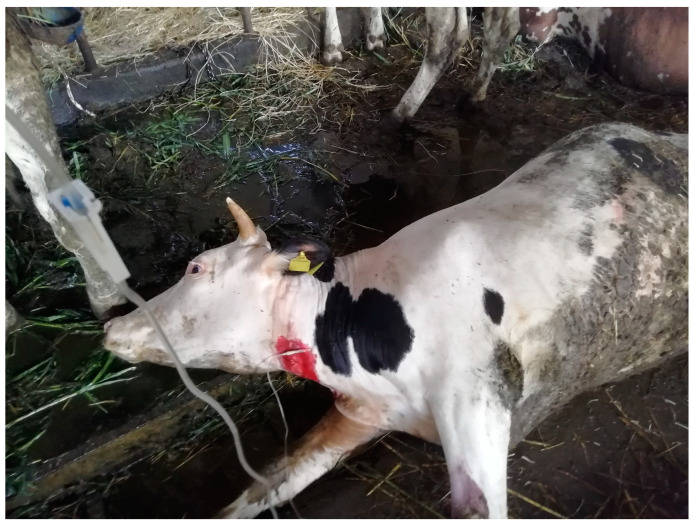
In Moretta (case B), a poisoned cow receiving the antidote (sodium thiosulphate) i.v. Note the cherry-red blood on the neck of the animal.

Owing to the similarity to the clinical picture described for the Sommariva del Bosco poisoning (case A) and based on the first analytical results revealing the mass presence of dhurrin in sorghum samples from that case, antidotal therapy was immediately started. However, due to the limited availability of sodium thiosulphate (Na_2_S_2_O_3_), it was decided to treat only the most severely affected individuals (*n* = 5), lying in sternal/lateral recumbency with panting and vocalisations. Antidote solution was prepared by dissolving 5 g Na_2_S_2_O_3_ in 4 L of Ringer’s lactate, which was slowly administered i.v. (Figure 4). 

Furthermore, 15 g of Na_2_S_2_O_3_ was dissolved in 10 L of cold water and then given orally through drench guns. At 10–15 min after antidote administration, breathing started to improve, and vocalisations almost ceased; the cows were again able to stand in about one hour.

#### 2.5.3. Case C

As mentioned above, poisoning symptoms were noticed during the night, and this resulted in difficulties in obtaining Na_2_S_2_O_3_ in sufficient amounts to treat all the affected animals (*n* = 30). It was therefore decided to administer methylene blue i.v. (10 g dissolved in 4 L of Ringer’s lactate) first; however, this treatment was only partially effective in reducing the severity of the clinical signs. As soon as Na_2_S_2_O_3_ was fully available (late in the morning), it was promptly administered i.v. (5 g dissolved in 4 L rehydrating solution) to all previously treated cows. This led to a rapid improvement of the clinical picture as described for case B. Twenty-eight cows survived, while two died few days later.

#### 2.5.4. Case D

No treatment was performed.

#### 2.5.5. Case E

Due to the alert system set up for the purpose of tackling the cyanogenic glycoside outbreaks, the antidote Na_2_S_2_O_3_ was made readily available to veterinarians. Accordingly, all poisoned animals were treated with the antidote as soon as 1 h after the onset of clinical signs, and a rapid recovery ensued within 2 h from therapeutic intervention. The treatment schedule was the one detailed for case B.

## 3. Results

### 3.1. Gross Lesions

Necropsy was performed on six carcasses: three from case A, one from case B and two from case C.

**Figure 5:**
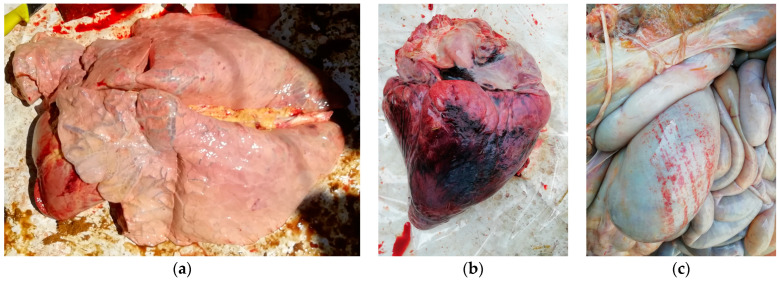
Necropsy findings in Moretta case (B), revealing lung emphysema (**a**), myocardial haemorrhages (**b**) and abomasitis (**c**).

On post mortem examination, an intense sweet smell of bitter almonds was reported. The blood was bright red and clotted poorly. The tracheas were congested, with oedema, petechiae and a variable amount of froth; severe pulmonary emphysema and oedema were also noticed (Figure 5a). Hydropericardium, focal haemorrhages and necrosis of the myocardium were observed (Figure 5b). The rumens were filled with fresh green material and bloated; suffusion and petechiae were present on the rumens, reticula and omasa as well. Congestion and petechial haemorrhages were observed in the gastrointestinal tracts. Finally, abomasitis (Figure 5c), severe splenomegaly, and enlarged and congested livers were observed in most of the animals. In a carcass from case C belonging to a cow that underwent the antidotal therapy but died two days after treatment, subcutaneous gelatinous necrosis was additionally detected.

### 3.2. Histopathological Lesions

In most of the animals, the pulmonary parenchyma revealed foci of alveolar oedema, emphysema and congestion of capillaries. Histologically, type I pneumocyte necrosis and hyperplasia of type II pneumocytes with hyaline membranes were observed, along with thickening of septa due to mononuclear cell infiltration. Three individuals presented hearts with large haemorrhagic areas in myocardial and pericardial adipose tissue. Focal fibrosis and moderate multifocal non-purulent myocarditis were also observed.

In one cow, which died two days after treatment (case C), the abomasum revealed acute abomasitis characterised by severe hyperaemia, red–brown haemorrhagic exudate adherent to the mucosa and neutrophilic infiltration with focal oedematous–haemorrhagic fluid in the submucosa. Thrombosis and regressive epithelial alterations were also observed in the mucosa.

### 3.3. Dhurrin Determination

Sorghum samples were taken from the farms involved in poisoning outbreaks and from other selected farms and fields, as detailed in Materials and Methods.

Table 2 reports the results related to the five intoxication cases. In case D, two samples from different areas of the pasture were collected. In case E, three samples from distinct parts of the plants and stages of maturity were tested. HCN concentrations are expressed in mg/kg; values over 200 mg/kg are generally considered dangerous [6,28]. In all but one case, Sorghum HCN concentrations were higher than 200 mg/kg; only in case E were there no samples that reached 200 mg/kg. Additionally, in one sample picked on the border of the pasture from case D, the HCN concentration measured 9 mg/kg.

One sample of ruminal content from a dead cow (case A) was also tested for dhurrin content with a negative result.

Dhurrin and HCN concentrations from the two farms selected for cultivated forage Sorghum are reported in Table 3 (Case A—Sommariva del Bosco) and Table 4 (Montechiaro d’Asti). For both farms, HCN content remained high for the whole period of August, tended to decline toward tolerable levels in September and reached negligible levels only in fall.

Taking the results together, no clear differences in HCN concentrations between different portions of the plants were noticed, with the exception of the culm. Indeed, in Montechiaro d’Asti, two culm samples collected on the 30th of August revealed lower HCN concentrations (0 and 147 mg/kg, respectively) than leaves and inflorescences (*n* = 7), which displayed HCN values in the range of 155–868 mg/kg. additionally, no strong evidence of a higher HCN content was found in younger/shorter individuals with respect to older/taller ones, even though two samples picked from ensiled bales on the 17th of November revealed a low HCN level (19 mg/kg) in a bale made of short immature plants (height < 50 cm) and no HCN at all in a bale made of mature individuals (height > 150 cm).

Concurrently, a set of samples of *S. halepense* were gathered from several farms and fields scattered across Cuneo, Asti and Torino provinces. Their dhurrin and HCN concentrations are listed in Table 5. Despite the close proximity of some of the sampling sites to farms experiencing poisoning cases from cyanogenic glycosides, only in one case was the threshold of concern of 200 mg/kg HCN reached, even in specimens collected in August. The frost-covered sample collected on the 23rd of November in Sampeyre, in a mountain area, revealed dhurrin concentrations <LOQ. In addition, a negative result was found in a sample from case D made of mixed grasses.

**Figure 6:**
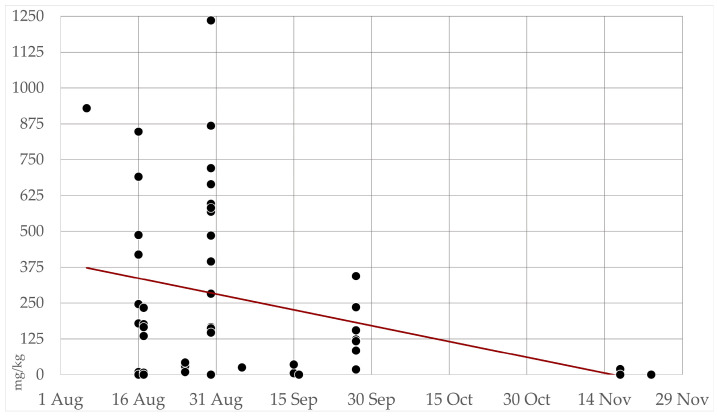
Seasonal trend of hydrogen cyanide (HCN) concentrations in all cultivated and wild collected Sorghum samples. Figure shows aggregate data (*n* = 57) from all samples collected in Piedmont, including all areas, from August to November 2022.

The seasonal trend of cyanide concentrations in all collected samples (*n* = 57) of either cultivated or wild Sorghum is depicted in Figure 6. Overall, a clear decreasing trend was noted: in August 2022, 58% of samples were found to contain levels > 200 mg/kg, whereas, from September to November, such amounts were detected in only 8% of the specimens.

## 4. Discussion

The rapid onset of clinical signs in cows shortly after the ingestion of Sorghum, followed sometimes by sudden death, had immediately suggested cyanide poisoning. Respiratory distress, stupor, sternal or lateral recumbency, convulsions, muscle tremors and sialorrhoea are typically reported in cyanide poisoning in cattle [6,29]. In addition, the recorded intense sweet odour of “bitter almonds”, the bright cherry-red colour of venous blood and the presence of lung congestion and emphysema as well as the presence of froth in the trachea are consistently recorded in bovines with cyanogenic glycoside poisoning [30]. The detection of abomasitis that features oedematous–haemorrhagic and neutrophilic granulocyte infiltrations has been also associated with cyanide poisoning [6]. Finally, myocardial haemorrhages further point to cyanide poisoning [31].

The gold-standard therapy for cyanide toxicosis [6,32] consists of supplying a chemical agent able to induce the formation of methaemoglobin (MetHb), i.e., oxidised (Fe^+++^) haemoglobin, which is unable to bind O_2_, and making it available to tissues. However, cyanide shows a higher affinity toward the Fe^+++^ central haem iron of MetHb than the Fe^+++^ of cytochrome oxidase. This causes the release of cyanide from the enzyme, the formation of cyanoMetHb and the reactivation of cell respiration. MetHb formation in large animals may be primarily accomplished by administering sodium nitrite i.v. (10 to 20 mg/kg bw); this treatment should be repeated with great care because of the danger of producing nitrite toxicosis, with further impairment of cellular respiration and severe hypotension [30]. Methylene blue at high dosages (1 to 3 g/~250 kg bw) has been recommended as an alternative to nitrites [31]. This treatment must be coupled with the sulphur donor Na_2_S_2_O_3_, which, in the presence of rhodanese, reacts with HCN, yielding thiocyanate (SCN^−^); this metabolite lacks any detrimental effects on cellular respiration and is rapidly excreted via the kidneys. In the reported cases herein, coupling methylene blue and Na_2_S_2_O_3_ administration did not seem to result in a visible improvement in therapeutic efficacy; a significant and rapid relief of the clinical signs was indeed obtained only after Na_2_S_2_O_3_ treatment, which was successfully used alone in cases B and E with 100% efficacy. It has actually been reported that, in cattle, there is no benefit of administering i.v. a MetHb-inducing agent over Na_2_S_2_O_3_ alone [32]. In addition, prompt oral dosing with Na_2_S_2_O_3_ may help in detoxifying HCN released in the rumen even before the onset of clinical signs [33]. The overall good success of the antidotal treatment further confirmed the diagnosis of cyanide poisoning. It should be noted that treated cows from case A had a relatively high survival rate (87%) even though they did not receive specific antidotes, but only a palliative fluid therapy with a multivitamin complex. The prompt removal of the animals from the contaminated pasture, i.e., after the first sudden deaths, was likely the cause of the high recovery rate.

According to the European Directive 2002/32/EC [34], a threshold of 50 mg/kg cyanide has been established for animal feed and raw materials. Under field conditions, concentrations over 200 mg/kg are considered sufficient to induce overt toxicosis [6,28,31].

It is generally assumed that crop plants are less resistant to parasites and herbivores than their wild counterparts due to artificial genetic selection aiming at reducing the content of specific defence compounds (e.g., cyanogenic glycosides) that may prove harmful for humans and livestock [35]. However, this assumption cannot be generalised to Sorghum. Unexpectedly, broomcorn cultivars such as Suzy (a *S. bicolor* × *S. sudanense* variety, Sommariva del Bosco, case A) and the mixture of Piper and Sudal (Montechiaro d’Asti), revealed very high HCN concentrations in August 2022. Both cultivars are specifically marketed for animal feeding purposes; however, guidelines for use reported on seeds’ envelopes do recommend not to feed the crop to animals when plants are below 70/80 cm (70 cm for the mixture of Piper and Sudal, and 80 cm for Suzy), but they lack any information on the potential related danger [36]. In case A, the farmer decided to allow his herd to graze on the field although the sorghum plants were below the recommended height. As was true for many other farmers during that summer, his farm was experiencing a shortage of forage due to its high cost and the scarce availability of green pastures. The increase in forage prices was a direct consequence of a lower supply on the market that, in turn, was caused by a diffuse drought particularly affecting Northwest Italy. A parallel survey was conducted on cultivated hybrids (*S. bicolor* ssp*. Drummondii* Piper and *S. bicolor* × *S. sudanense* Sudal) from different fields surrounding a farm in the Asti province (Montechiaro d’Asti) near poisoning case D; HCN concentrations >200 mg/kg were detected in 50% of specimens collected in August 2022, with peaks of 847–868 mg/kg. Overall, our findings confirm that bovines should not be fed on young plants even of cultivated hybrids, including regrowth after cutting, because of the high risk of cyanide poisoning.

In the outbreak of cyanogenic glycoside poisoning in cows described herein, *S. halepense* was implicated in 4 out 5 cases. Johnson grass is considered among the most invasive and dangerous weeds in Europe and extra-European countries; aside from the potential accumulation of toxic amounts of cyanogenic glycosides, several potentially adverse effects have been reported, including displacement of natural flora; competition with other crops; synthesis of allelochemicals interfering with crop growth; and hosting of plant pathogens (for a review, see Peerzada et al., 2017 [37], and the numerous literature references therein). Despite that, the free growth of Johnson grass is rarely counteracted; in fact, as reported in four cases (B to E), farmers traditionally employ Johnson grass as a fodder plant (hay or pasture) during periods of droughts. As with other Sorghum species, several factors, including soil chemical composition, plant age, use of nitrogen fertilisers, weather conditions and damage to plant tissues, are reported to affect the dhurrin content and hence the potential HCN release of Johnson grass [34]. There is scant information on the dhurrin and HCN content of *S. halepense*, particularly from European countries. In a study performed in India, calculated HCN concentrations (based on the colorimetric method) of uncultivated Johnson grass from farm bunds averaged around 900 mg/kg at 30 days after weeding but fell to 120 mg/kg at the 25% flowering stage [38]. Therefore, as with cultivated Sorghum species, cattle should not be fed with Johnson grass at the early stage of the crop. In the outbreaks reported here, three poisoning cases concerning *S. halepense* revealed HCN concentrations in the range 419–690 mg/kg (cases B, C, D). The relatively low amount of HCN (below 50 mg/kg) detected in plant specimens from case E is probably attributable to uncorrected sampling procedures. For comparison, samples of *S. halepense* were collected in a more scattered way during August and September 2022 in fields from farms located in different areas of Piedmont, even near poisoning cases (Sanfrè, Faule, Montiglio Monferrato); of note, only in one case were HCN amounts >200 mg/kg detected in plant specimens, likely pointing to the occurrence of different pedo-climatic conditions not resulting in remarkable accumulation of dhurrin as was reported for the areas of the outbreak.

As a matter of fact, in summer 2022, unfavourable weather conditions were registered all across Europe, and Northern Italy, particularly certain areas of Piedmont, resulted one of the driest regions [39]. According to the Piedmont Regional Agency for Environmental Protection (ARPA), summer 2022 was one of the hottest and driest of the last 30 years in Piedmont [23]. Indeed, during that summer, unprecedented temperatures were registered, occasionally reaching all-time highs (Figure S5). Additionally, the numbers of tropical nights (T > 20 °C) and days (T > 30 °C) were higher than in previous years (Table S2). Moreover, rainfalls were irregular in terms of both quantity and regional distribution, with a decrease of 50–60% with respect to previous years, especially in areas where cyanide poisoning outbreaks occurred (Figures S6 and S7). Finally, the hydric balance had been in deficit since the previous winter (Figure S8), also due to limited snow reserves. These conditions are reasonably believed to be responsible for the excessive accumulation of dhurrin observed in most *Sorghum* specimens collected in the outbreak area and surrounding areas.

## 5. Conclusions

The use of *Sorghum* plants for cattle feeding is a common practice in Northern Italy, where the most abundant species are the wild weed *S. halepense* and several cultivars of *S. bicolor*. Data from the described outbreaks in Piedmont indicate that not only Johnson grass but also different Sorghum cultivars may accumulate dhurrin concentrations that can trigger lethal poisoning of cows, particularly if animals are fed with young plants or sprouts and under adverse climatic conditions (drought, tropical temperatures). As expected, the decrease in diurnal and nocturnal temperatures together with the increase in the amount of precipitation occurring in October and November caused dhurrin levels to decline and, thus, HCN concentrations to fall well below the toxic threshold of 200 mg/kg.

Accurate management is needed when using Sorghum forages, and specific instructions should be reported on every commercial *Sorghum* seed envelope. Moreover, both farmer unions and local authorities should disseminate technical information on how to avoid toxicoses when feeding animals with cultivated Sorghum, including instructions/good practices for the safe use of *S. halepense* as a fodder. Finally, our data further support the use of sodium thiosulphate alone in the treatment of cyanogenic glycoside poisonings in cattle, suggesting that this antidote should be made readily available to veterinary practitioners in order to ensure a rapid and efficacious intervention.

## Figures and Tables

**Table 1:** Treatments given, n. of surviving animals and therapeutical success rate in the described outbreak of Sorghum toxicosis. Case D is not included because cows were not subjected to any treatment.

Case	Type of Treatment	Treated Animals	Surviving Animals	Success Rate
A	Rehydrating solutions + multivitamin complex	30	26	87%
B	Sodium thiosulphate	5	5	100%
C	Methylene blue and (later) sodium thiosulphate	30	28	93%
E	Sodium thiosulphate	40	40	100%

**Table 2:** Dhurrin and hydrogen cyanide (HCN) concentrations in *Sorghum* samples implied in the five outbreaks of cyanogenic glycoside poisoning occurring in Piedmont in August 2022. When plant part is not specified, analysis was performed on the whole plant.

Case	Date	Sorghum Species	Site of Collection	Dhurrin (mg/kg)	HCN (mg/kg)
A	6-Aug	Suzy ^1^ (sprout, height 30–45 cm)	Pasture	10.717	929
B	16-Aug	*S. halepense*	Trough	5.627	487
C	16-Aug	*S. halepense*	Trough	7.961	690
D	16-Aug	*S. halepense*	Pasture	4.834	419
		*S. halepense*	Pasture border	104	9
E	25-Aug	*S. halepense* (young plants)	Pasture	335	29
		*S. halepense* (leaf mix)	Pasture	488	42
		*S. halepense* (inflorescence)	Pasture	105	9

^1^ *S. bicolor* × *S. sudanense* cultivar called Suzy.

**Table 3:** Time course of dhurrin and hydrogen cyanide (HCN) concentrations in the *Sorghum bicolor* × *Sorghum sudanense* variety called Suzy from Sommariva del Bosco (case A). When plant part is not specified, analysis was performed on the whole plant.

Date	Dhurrin (mg/kg)	HCN (mg/kg)	Notes
6-Aug	10,717	929	Sample related to the outbreak (case A)
14-Aug	6869	596	Average plant height 50 cm
16-Aug	14,246	1235	Open-air dried
16-Aug	5590	485	Fresh, leaves > 1 m
16-Aug	8300	720	Fresh, leaves < 50 cm
17-Aug	<LOQ	0	Bundled; cut on the 14th of July
21-Aug	6550	568	Average plant height 60 cm
27-Aug	7661	664	Average plant height 68 cm
5-Sept	1420	123	
12-Sept	1798	155	
23-Sept	958	83	
27-Sept	974	84	Field “Paolorio”
27-Sept	1354	117	Field “Luppiano”
27-Sept	2707	235	Field “Valè”
6-Oct ^1^	<LOQ	0	Fresh, chopped
23-Nov	<LOQ	0	Mature silage (45 days), from mixed fields

^1^ The same result (0 mg/kg HCN) was measured in six samples from 5 different fields.

**Table 4:** Time course of dhurrin and hydrogen cyanide (HCN) concentrations in *Sorghum* samples from a farm in the Asti province (Montechiaro d’Asti), located near case D. All the samples belong to mixed individuals grown from a seed mixture of two varieties: *S. bicolor* ssp*. drummondii* Piper and *S. bicolor* × *S. sudanense* Sudal. When plant part is not specified, analysis was performed on the whole plant.

Date	Dhurrin (mg/kg)	HCN (mg/kg)	Notes
16-Aug	9770	847	Sowed at the beginning of June, never cut
16-Aug	2840	246	Grown-back plants
16-Aug	2065	179	Sowed at the beginning of June, grazed in July
30-Aug	1792	155	Field 1; leaves > 150 cm
30-Aug	1919	166	Field 1; leaves ~ 50 cm
30-Aug	<LOQ	0	Field 1; culm
30-Aug	3251	282	Field 1; inflorescence
30-Aug	1865	162	Field 1; grown-back plants, without roots
30-Aug	10,010	868	Field 2; leaves > 150 cm
30-Aug	6701	581	Field 2; leaves ~ 50 cm
30-Aug	1697	147	Field 2; culm
30-Aug	4553	395	Field 2; inflorescence
26-Sept	3967	344	Culm and leaves
26-Sept	205	18	Inflorescence
17-Nov	229	19	Immature plants (without grains) ~ 50 cm; from ensiled bale
17-Nov	<LOQ	0	Mature plants (with grains) > 150 cm; from ensiled bale

**Table 5:** Dhurrin and hydrogen cyanide (HCN) concentrations in *Sorghum halepense* collected from different farms and fields in Piedmont during 2022. The analysis was performed on the whole plants.

Date	Location	Province	Dhurrin (mg/kg)	HCN (mg/kg)	Notes
16-Aug	Asti	Asti	<LOQ	0	Mixed grasses
17-Aug	Faule	Cuneo	85	7	Cut for haymaking
17-Aug	Verrua Savoia	Torino	1558	135	Field used for haymaking
17-Aug	Montiglio M.to	Asti	2036	176	Field “Sant’Anna”
17-Aug	Montiglio M.to	Asti	2693	233	Field “Acquedotto”
17-Aug	Montiglio M.to	Asti	1917	166	Field “Vallone”
5-Sept	Bra	Cuneo	289	25	Field of case C; forage for silo
15-Sept	Cuneo	Cuneo	57	5	Plants > 50 cm
15-Sept	Cuneo	Cuneo	401	35	Plants < 50 cm
16-Sept	Sanfrè	Cuneo	<LOQ	0	Mature plants (with inflorescence)
23-Nov	Sampeyre	Cuneo	<LOQ	0	Frost-covered plants

## Data Availability

Data are contained within the article.

## Supplementary Materials

The following supporting information can be downloaded at: https://www.mdpi.com/article/10.3390/ani14050743/s1, Table S1: LC–MS/MS parameters for dhurrin determination; Table S2: Numbers of tropical days (T > 30 °C) and nights (T > 20 °C) measured in every province of Piedmont in 2022; Figure S1: Calibration curve of dhurrin; Figure S2: Representative chromatograms of dhurrin reference material at 2.5 µg/mL; Figure S3: Representative chromatograms of a Sorghum sample; Figure S4: Representative chromatograms of a blank Sorghum sample; Figure S5: Daily temperature anomaly in Piedmont during the summer 2022 compared to the period 1991–2020; Figure S6: Rainfall anomaly (%) in Piedmont basins during summer 2022 compared to the period 1991–2020; Figure S7: Rainfall anomaly (%) in Piedmont basins during September 2022 compared to the period 1991–2020; Figure S8: Daily hydro-climatic balance (i.e., the difference between rainfall and evapotranspiration expressed in mm) in Piedmont in 2022 compared to the period 1959–2022. Data in Table S2 and Figures S5–S8 were extracted from the 2022 drought report, published by the Piedmont Regional Agency for Environmental Protection (ARPA) [23], openly available at https://www.arpa.piemonte.it/news/pubblicato-il-rappporto-sulla-siccita2019-in-piemonte-nel-2022 (accessed on 22 November 2023).
