# Supplementary material for: Overview of Cyanide Poisoning in Cattle from Sorghum halepense and S. bicolor Cultivars in Northwest Italy
Source: Animals (Basel). 2024 Feb 27;14(5):743. doi: 10.3390/ani14050743 (PMC10930883; doi:10.3390/ani14050743)

Figure S1. Calibration curve of dhurrin.

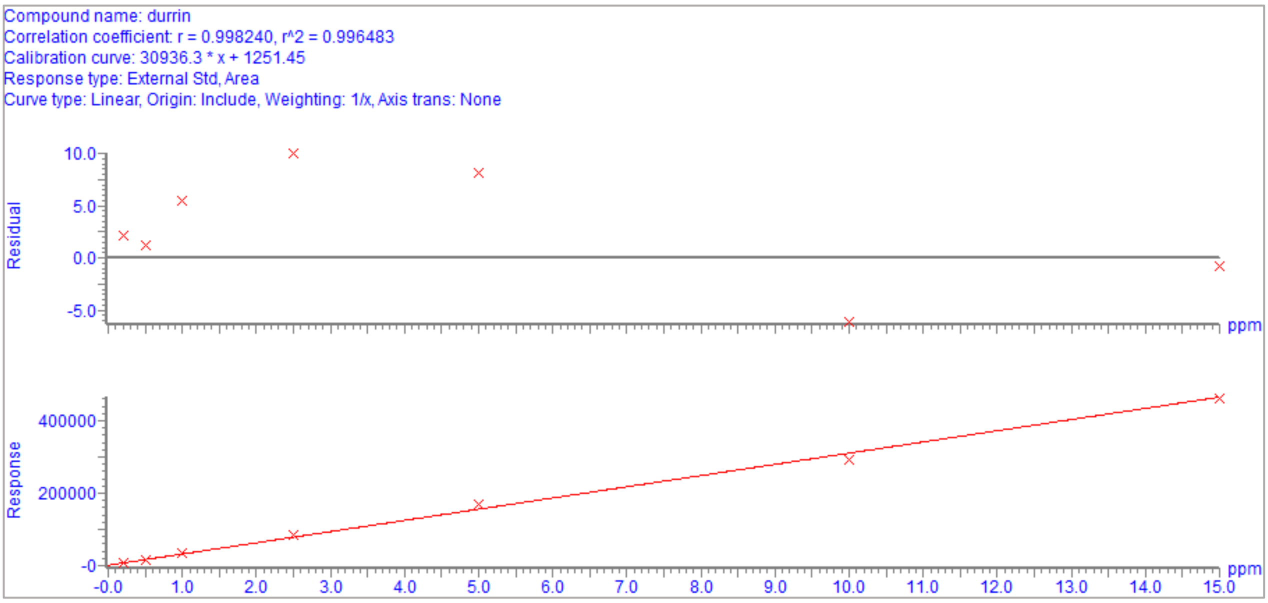

Figure S2. Representative chromatograms of dhurrin reference material at 2.5 µg/mL.

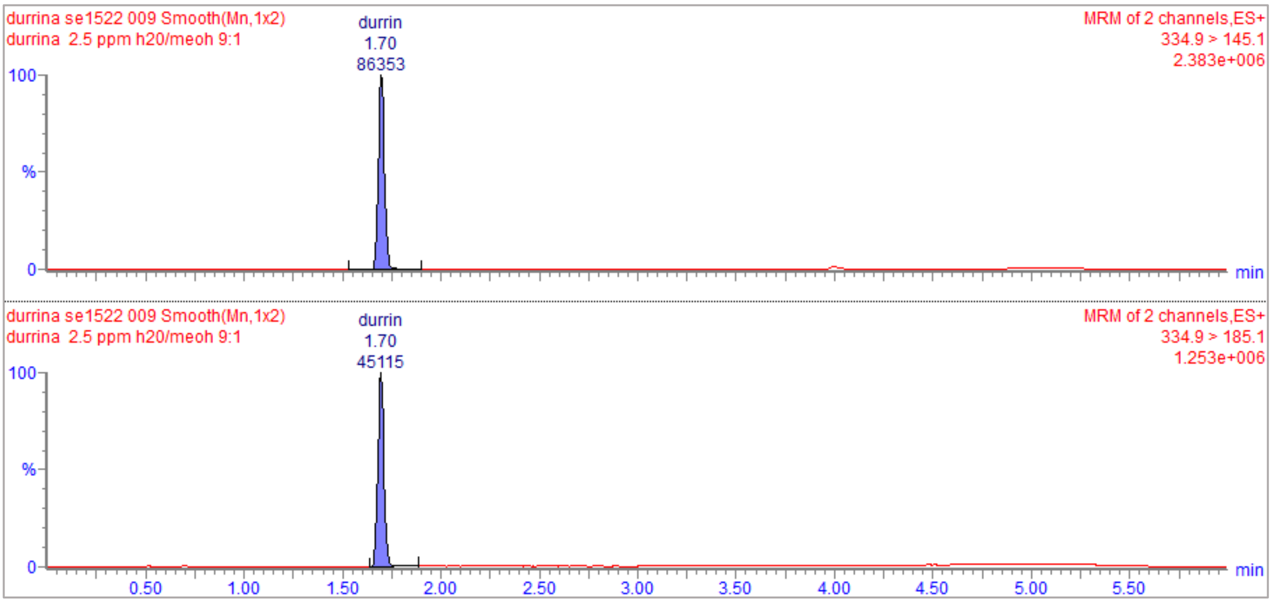

Figure S3. Representative chromatograms of a contaminated Sorghum sample (case A, Sommariva del Bosco).

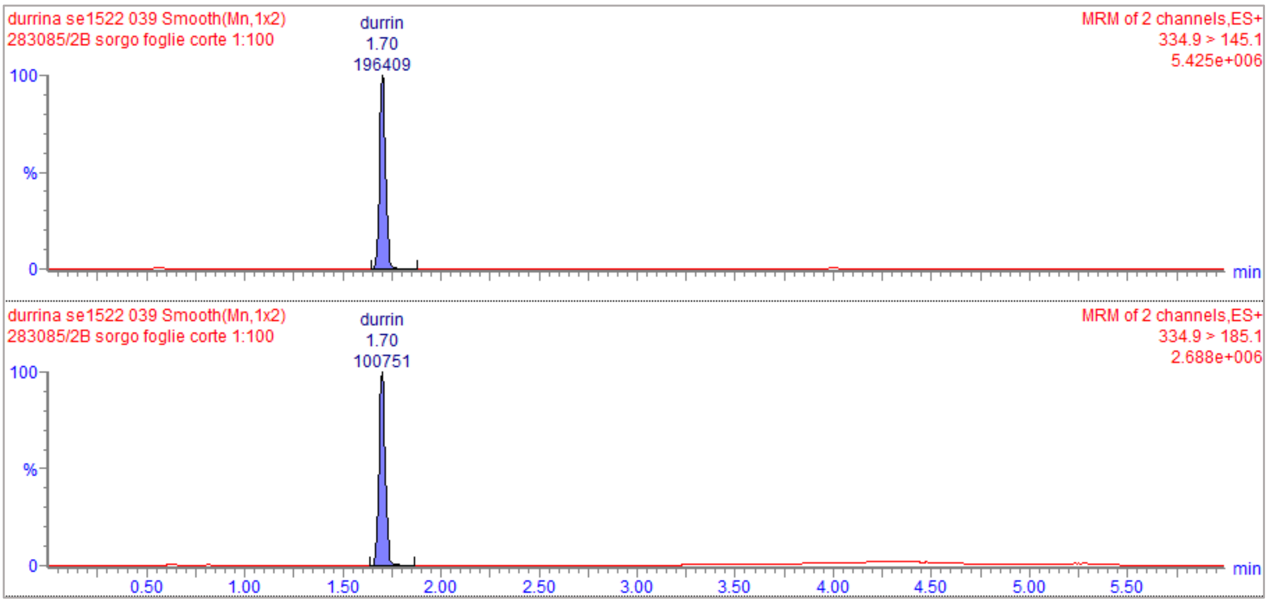

Figure S4. Representative chromatograms of a blank Sorghum sample.

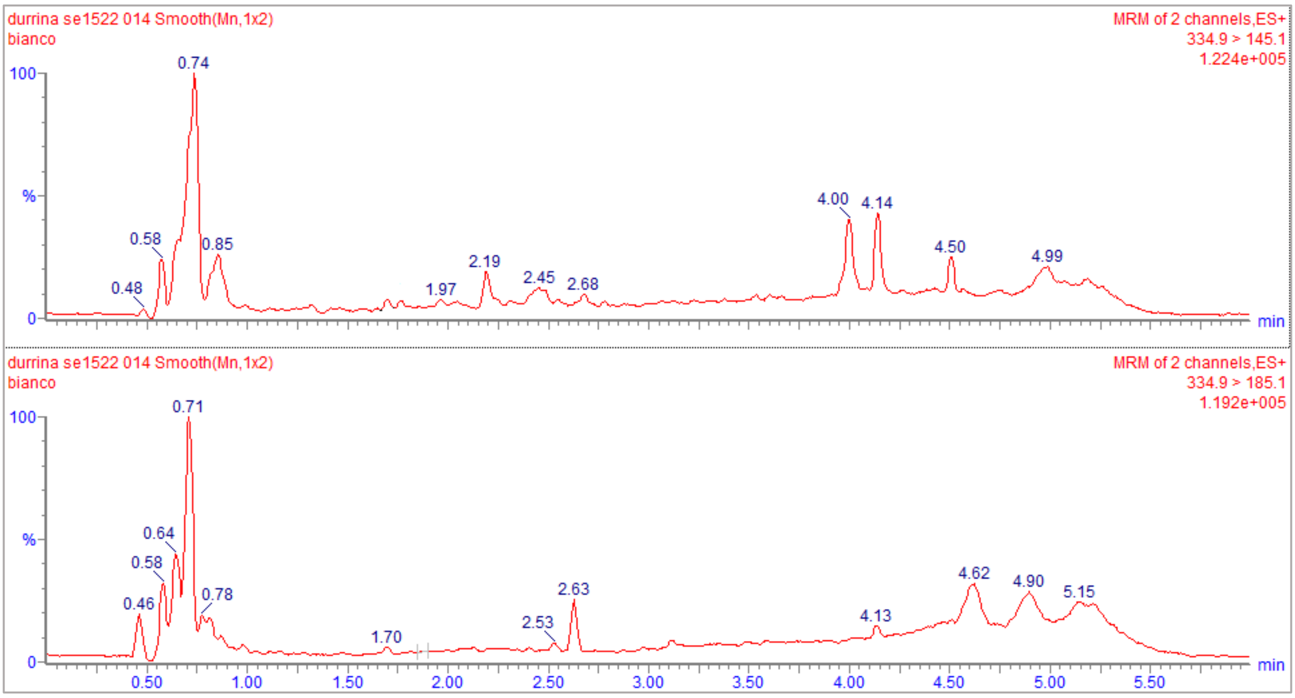

Figure S5. Daily temperature anomaly in Piedmont during the summer 2022 compared to the period 1991-2020. Black line indicates 2022 temperatures; red areas indicate values above the average of the period 1991-2020; blue areas the values below the average. 5- and 95-percentile are shown (dotted lines). Historical highs and lows (purple and green dotted lines, respectively), 5th and 95th percentile (yellow area) are also shown.

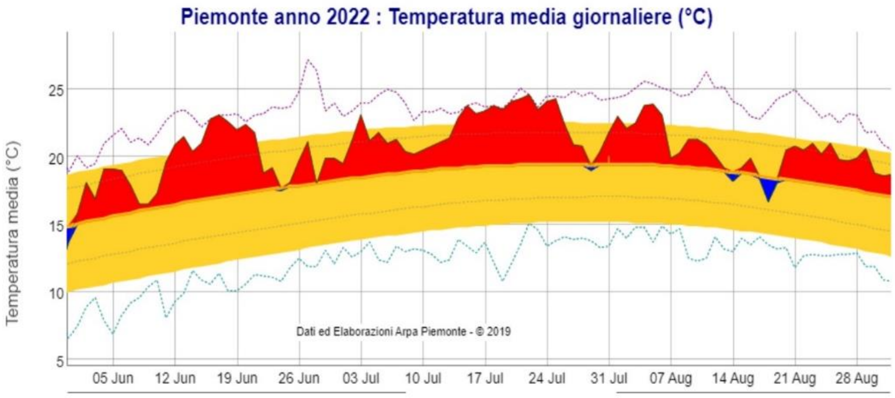

Figure S6. Rainfall anomaly (%) in Piedmont basins during summer 2022 compared to the period 1991-2020. Increasing reddish colouring highlights strong anomaly/decrease; increasingly bluish/purplish indicates an increase in rainfall.

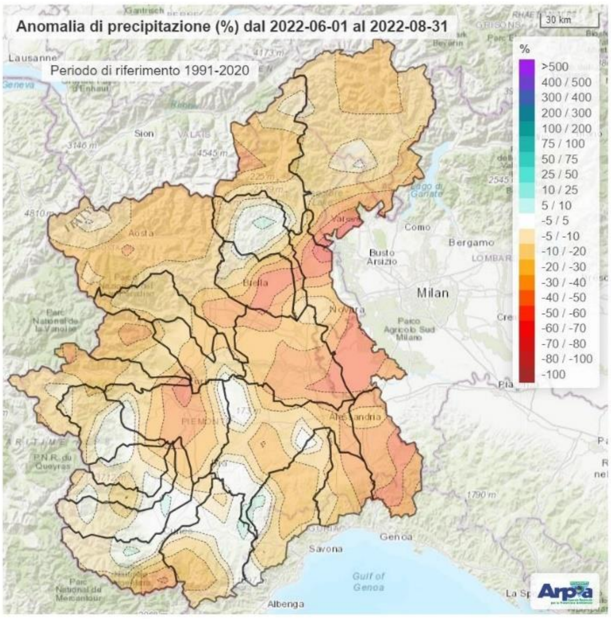

Figure S7. Rainfall anomaly (%) in Piedmont basins during September 2022 compared to the period 1991-2020. Increasing reddish colouring highlights strong anomaly/decrease; increasingly bluish/purplish indicates an increase in rainfall.

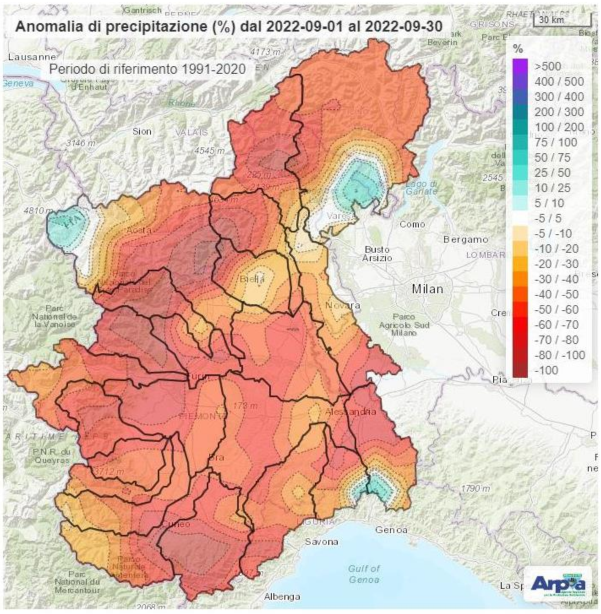

Figure S8. Daily hydro-climatic balance (i.e. the difference between rainfall and evapotranspiration expressed in mm) in Piedmont in 2022 compared to the period 1959-2022. Black line: 2022 hydro-climatic balance. Measures of central tendency of the period 1959-2022 are shown: 5th percentile (red dotted line); first quartile (yellow dotted line); average (green line); third quartile (blue dotted line); 95th percentile (purple dotted line).

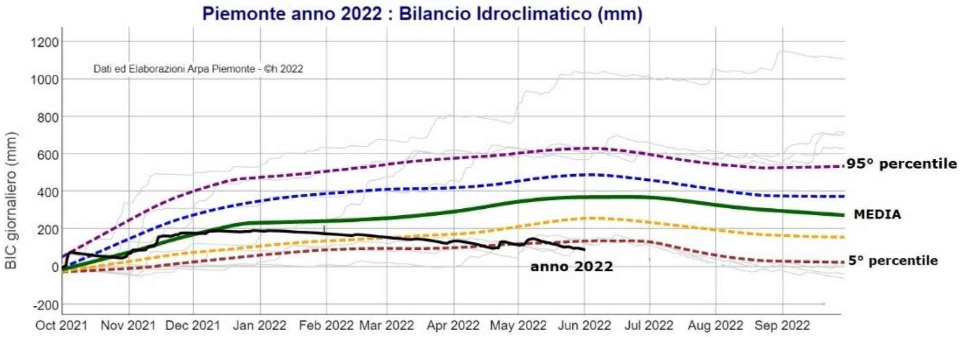

Supplement: Supplementary file 1 [file animals-14-00743-s001.zip › Supplementary figures.pdf]
