# Supplementary material for: Overview of Cyanide Poisoning in Cattle from Sorghum halepense and S. bicolor Cultivars in Northwest Italy
Source: Animals (Basel). 2024 Feb 27;14(5):743. doi: 10.3390/ani14050743 (PMC10930883; doi:10.3390/ani14050743)
Supplement: Supplementary file 1 [file animals-14-00743-s001.zip › Supplementary tables.pdf]

Table S1. LC-MS/MS parameters for Dhurrin determination.

Detection						
Analyte	Retention time	Precursor ion $[M+H]^+$ (m/z)	Dwell time (s)	Cone (V)	Collision Energy (eV)	Daughter ions (m/z)
Dhurrin	1.7	334.9	0.163	34	12	145.1 (Q*)
				34	10	185.1

Table S2. Numbers of tropical days (T > 30°C) and nights (T > 20°C) measured in every province of Piedmont in 2022. Data are compared to the average numbers of the period 1991-2020.

	**Tropical Days**		**Tropical Nights**	
	**1991-2020**	**2022**	**1991-2020**	**2022**
Alessandria	55.8	77	2.9	11
Asti	49.6	72	4.3	13
Biella	33.2	55	28.4	49
Cuneo	15.7	28	0.9	4
Novara	39.7	57	32.5	55
Torino	44	79	17.2	45
Verbania	38.7	61	19.9	45
Vercelli	45.9	65	8.6	28
